# Recent advances in the crosstalk between adipose, muscle and bone tissues in fish

**DOI:** 10.3389/fendo.2023.1155202

**Published:** 2023-03-14

**Authors:** Isabelle Hue, Encarnación Capilla, Enrique Rosell-Moll, Sara Balbuena-Pecino, Valentine Goffette, Jean-Charles Gabillard, Isabel Navarro

**Affiliations:** ^1^ Laboratory of Fish Physiology and Genomics, UR1037, Institut National de Recherche pour l'Agriculture, l'Alimentation et l'Environnement (INRAE), Rennes, France; ^2^ Departament de Biologia Cel·lular, Fisiologia i Immunologia, Facultat de Biologia, Universitat de Barcelona, Barcelona, Spain

**Keywords:** inter-tissue communication, adiponectin, leptin, TNF, TGFs, myostatin, IGFs, osteocalcin

## Abstract

Control of tissue metabolism and growth involves interactions between organs, tissues, and cell types, mediated by cytokines or direct communication through cellular exchanges. Indeed, over the past decades, many peptides produced by adipose tissue, skeletal muscle and bone named adipokines, myokines and osteokines respectively, have been identified in mammals playing key roles in organ/tissue development and function. Some of them are released into the circulation acting as classical hormones, but they can also act locally showing autocrine/paracrine effects. In recent years, some of these cytokines have been identified in fish models of biomedical or agronomic interest. In this review, we will present their state of the art focusing on local actions and inter-tissue effects. Adipokines reported in fish adipocytes include adiponectin and leptin among others. We will focus on their structure characteristics, gene expression, receptors, and effects, in the adipose tissue itself, mainly regulating cell differentiation and metabolism, but in muscle and bone as target tissues too. Moreover, lipid metabolites, named lipokines, can also act as signaling molecules regulating metabolic homeostasis. Regarding myokines, the best documented in fish are myostatin and the insulin-like growth factors. This review summarizes their characteristics at a molecular level, and describes both, autocrine effects and interactions with adipose tissue and bone. Nonetheless, our understanding of the functions and mechanisms of action of many of these cytokines is still largely incomplete in fish, especially concerning osteokines (i.e., osteocalcin), whose potential cross talking roles remain to be elucidated. Furthermore, by using selective breeding or genetic tools, the formation of a specific tissue can be altered, highlighting the consequences on other tissues, and allowing the identification of communication signals. The specific effects of identified cytokines validated through *in vitro* models or *in vivo* trials will be described. Moreover, future scientific fronts (i.e., exosomes) and tools (i.e., co-cultures, organoids) for a better understanding of inter-organ crosstalk in fish will also be presented. As a final consideration, further identification of molecules involved in inter-tissue communication will open new avenues of knowledge in the control of fish homeostasis, as well as possible strategies to be applied in aquaculture or biomedicine.

## 1 Introduction

Our understanding of the relationship and communication between skeletal muscle, bone and adipose tissue has undergone a major resurgence in the past decade, with an increasing number of published papers on the subject, especially in mammalian models (1). Nonetheless, a growing interest in fish species exists considering they are the largest and most diverse group of vertebrates. Nowadays, skeletal muscle, bone and adipose tissue are no longer viewed from the simple mechanical and metabolic perspective. These tissues can produce a high variety of molecules with autocrine, paracrine, and endocrine functions that mediate a complicated network of cross talking to communicate each other their specific physiological status. Moreover, lipid metabolites, either *de novo* synthesized or derived from dietary fats, named lipokines, could also act as signaling molecules between organs to coordinate energy substrates’ use (2).

Some of the earliest and current evidence for tissues crosstalk comes out from the literature involving the role of cytokines in different human pathologies and during physical activity (3, 4). The use of zebrafish (*Danio rerio*) as an advantageous animal model in biomedicine, together with the increasing interest in the integrative knowledge of endocrine control of growth and metabolism in cultured fish species, has focused the attention to the crosstalk between bone, muscle, and adipose tissues in piscine models. Nevertheless, the information in fish inter-tissue communication is still limited compared with that of mammals.

Based on this background, the aim of this review is to give an overview about the most up-to-date knowledge in the field of adipokines, myokines and osteokines in fish, and their possible roles in tissues crosstalk.

## 2 Adipokines

**Figure 1:**
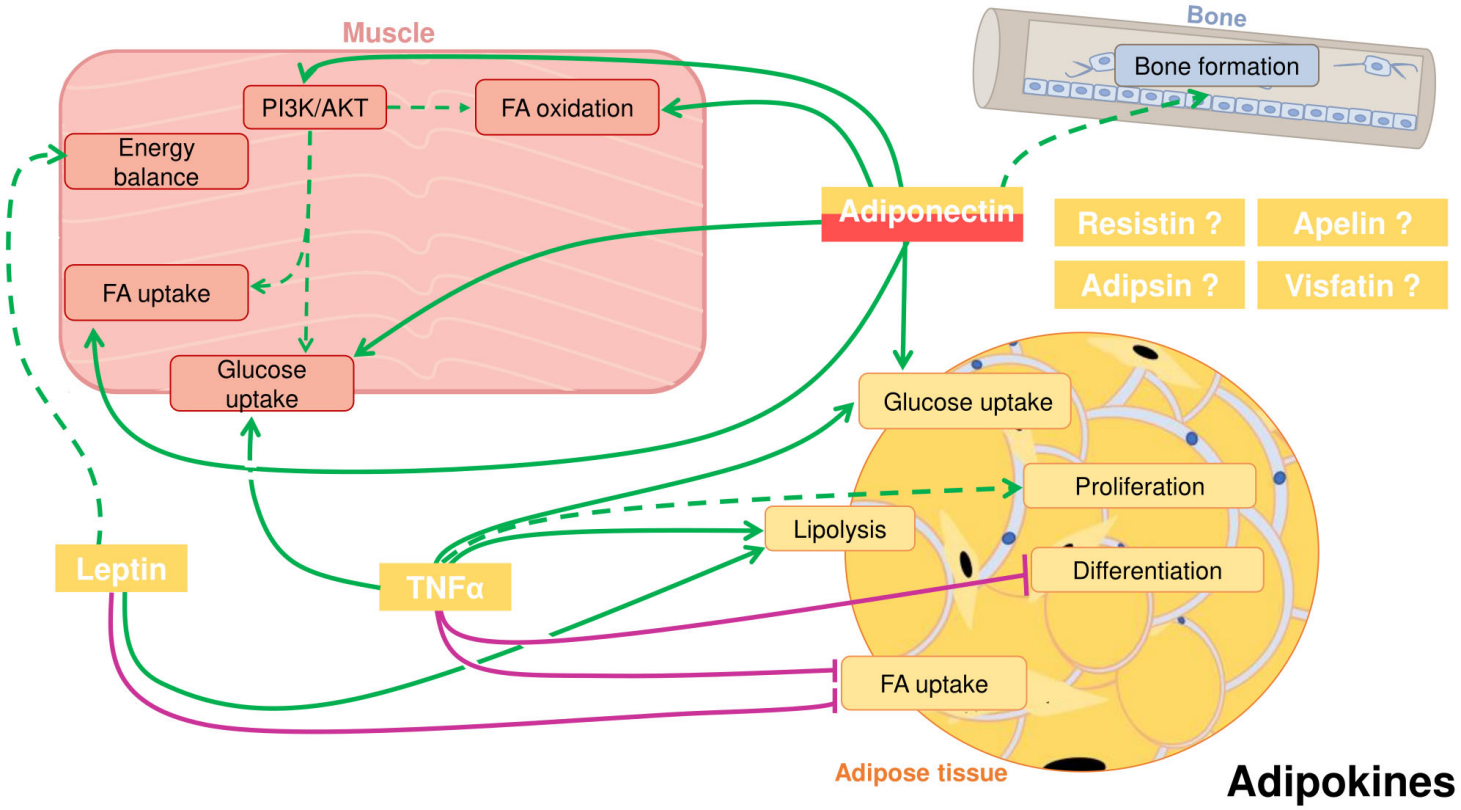
Crosstalk between adipose tissue, muscle, and bone in fish through cytokines secreted by the adipose tissue, also known as adipokines. Adiponectin, that can also be secreted by skeletal muscle, can positively influence in muscle fatty acid (FA) and glucose uptake, FA oxidation and activate the PI3K/AKT pathway. Studies suggest a role for adiponectin inducing bone formation. Adiponectin has also autocrine functions in the adipose tissue, where it can promote glucose uptake. Leptin in muscle can presumably influence energy balance. In the adipose tissue, leptin induces lipolysis and inhibits FA uptake. Tumor necrosis factor α (TNFα) can stimulate glucose uptake in muscle and has an important autocrine role in adipose tissue. There, TNFα stimulates glucose uptake and lipolysis, and probably adipocyte proliferation, whereas it inhibits differentiation and FA uptake. Other potential adipokines are resistin, apelin, adipsin and visfatin. Positive proved effects: green solid line; Positive suggested effects; green dotted line; Negative proved effects: purple solid line.

The main function of vertebrate adipose tissue is the storage of triglycerides under conditions of excess of calories and their release during periods of energy demand. In addition, adipose tissue is now recognized as a complex endocrine organ. Adipose tissue is primarily composed of adipocytes, as well as pre-adipocytes, stem cells, endothelial cells, or macrophages, which contribute to the release of metabolites, lipids, and bioactive peptides, so-called adipokines. These secreted factors can regulate the tissue locally, but also many other tissues, thus controlling the whole organism’s physiology. In this section, we will focus on the best-known adipokines in teleost fish, keeping in mind their described effects in the adipose tissue itself, but also at a systemic level, including the regulation of different biological processes especially in muscle and bone as target organs (**Figure 1**; **Table 1**). Nevertheless, it is important to note that our understanding of the function and mechanisms of action of many of these adipokines is still largely incomplete in fish.

**Table 1:** List of reported adipokines and myokines in fish with summarized function in the crosstalk between tissues.

Factor	Approach	Target tissue	Species	Function	Reference
Adiponectin	Recombinant human globular adiponectin. Expressed in adipose tissue and muscle	Primary myocytes	Rainbow trout (*Onchorynchus mykiss*)	Activated the PI3K/Akt pathway, which was inhibited by pre-incubation with wortmannin in myotubes (day 11 of culture) Increased FA oxidation, reported as CO_2_ production Reduction in the FA in the medium in myotubes (day 11 of culture)	(5)
	Recombinant human globular adiponectin	Primary adipocytes	Rainbow trout (*Onchorynchus mykiss*)	Increased glucose uptake in adipocytes at day 15 of culture	(6)
	Changes in plasma	Bone tissue	Zebrafish (*Danio rerio*)	Diet-induced obese model presented decreased adiponectin levels parallel to an osteoporotic-like phenotype, characterized by reduced scales mineralized area and ALP activity, and increased TRAP activity	(7)
	Recombinant large yellow croaker adiponectin	Primary myocytes	Large yellow croaker (*Larimichthys crocea*)	In cell suspensions: Increased gene expression of *adipor1*, *adipor2*, *appl1*, *lkb1* and *ampkα*, transcription factors (*rxr*, *pparγ* and *srebp1*) and genes related to FA oxidation (*cpta*, *cptb* and *aco*), synthesis (*scd1*, *elovl4* and *elovl5*) and uptake (*cd36*, *fabp10* and *fabp11*) Increased protein levels of APPL1, p-AMPKα and PPARγ Promoting effects were blocked by knockdown of *appl1* and *adipors*	(8)
Leptin	Recombinant trout leptin	Primary adipocytes	Rainbow trout (*Onchorynchus mykiss*)	Downregulated expression of *lpl* and *fatp1* and increased lipolysis, measured by glycerol release in mature isolated adipocytes	(9)
	Leptin expression and secretion	Primary adipocytes	Rainbow trout (*Onchorynchus mykiss*)	Leptin expression and release increased from pre-adipocytes to mature adipocytes in culture (day 7 to day 16)	(9)
	Knockout *lepr*	Skeletal muscle	Zebrafish (*Danio rerio*)	Knockout of leptin receptor gene (*lepr*) in a hepatocellular carcinoma model induced a higher survival rate and lower muscle-wasting level	(10)
	Recombinant trout leptin	Primary adipocytes	Gilthead sea bream (*Sparus aurata*)	Inhibited lipid accumulation Decreased gene expression of *pparγ* and *cd36* at early (day 8) and late (day 12) stages of cell differentiation	(11)
Resistin	Expression in liver		Siberian sturgeon (*Acipenser baerii*)	Hepatic *resistin* expression is affected by nutritional status	(12)
TNFα	Recombinant human TNFα	Primary adipocytes	Rainbow trout (*Onchorynchus mykiss*)	Lipolytic effect *via* protein kinases, upregulated *lxr* expression and downregulated adipor2 expression in isolated adipocytes. Enhanced proliferation at low dose and inhibited adipocyte differentiation (days 5 and 7). Inhibited insulin-induced increase in *adiponectin* mRNA levels (day15)	(5, 6, 13–15)
	Recombinant human TNFα	Primary adipocytes and myocytes	Rainbow trout (*Onchorynchus mykiss*)	Stimulated glucose uptake in myoblasts (day 2) and myotubes (day 10). Downregulated *adipor1* and *adiponectin* expression in myotubes (day 11 of culture)	(5, 16)
	Recombinant human TNFα	Primary adipocytes	Large yellow croaker (*Larimichthys crocea*)	Decreased *atgl* expression in pre-adipocytes. Decreased FA uptake and fat content downregulating *lpl* expression and increased *pparα* and *pparγ* expression during lipolysis in differentiated adipocytes. At high concentrations, inhibited adipocyte proliferation along the culture	(17)
	Recombinant seabream TNFα	Primary adipocytes	Gilthead sea bream (*Sparus aurata*)	Downregulated *pparγ* and *pparb* expression and promoted lipolysis in mature isolated adipocytes	(18, 19)
Myostatin	Expression in muscle		Barramundi (*Lates calcarifer*)	A fasting period upregulated *mstn-1* but not *mstn-2* expression in muscle and liver but decreased *mstn-1* expression in brain and gills	(20)
	Recombinant human MSTN	Primary myocytes	Rainbow trout (*Onchorynchus mykiss*)	Inhibited myoblast (day 1) proliferation and induced myotube atrophy. Did not decreased MyoD and Myogenin protein levels, so had no effect on myoblast (day 2) differentiation	(21, 22)
	Inactivation of *mstn* gene	Muscle tissue	Medaka (*Oryzias latipes*)	Inactivation of *mstn* gene increased 25-30% of muscle mass with some alterations in the immune system	(23, 24)
	Knockout of *mstnb* gene	Muscle and adipose tissue	Zebrafish (*Danio rerio*)	Inactivation of *mstnb* but not *mstn*a, increased muscle mass after 80 days post fertilization associated to muscle hyperplasia. Increased fat accumulation in muscle of *mstnb* -/- and the energy supply from an amino acid-dependent source is exchanged for a lipid-dependent source	(25, 26)
IGF-1 and IGF-2	Recombinant trout IGF-1	Primary myocytes	Rainbow trout (*Onchorynchus mykiss*)	Stimulated glucose and L-alanine uptake, and cell proliferation ([^3^H]-thymidine uptake) at day 4 and 10 of culture development	(27)
	Recombinant human IGF-1 and IGF-2	Primary myocytes	Rainbow trout (*Onchorynchus mykiss*)	Enhanced glucose uptake by both IGFs at days 4, 7 and 10 of culture Increased cell proliferation by IGF-2, reported as [^3^H]-thymidine incorporation Activation of the MAPK pathway by phosphorylation of the ERK1/2 protein by both IGF-1 and IGF-2 at days 4 and/or 11 Stimulated Akt phosphorylation by IGF-1 and IGF-2 at days 4 and 11 of culture	(28)
	Recombinant salmon IGF-1 and IGF-2	Primary myocytes	Atlantic salmon (*Salmo salar*)	Stimulated *igf-1* and *igfbp6* mRNA levels by both IGFs	(29)
	Recombinant rat IGF-1 and IGF-2	Primary myocytes	Rainbow trout (*Onchorynchus mykiss*)	Stimulated cell proliferation (BrdU labeling) by both IGF-1 and IGF-2 at day 4 of culture development	(30)
	Recombinant human IGF-1	Primary myocytes	Rainbow trout (*Onchorynchus mykiss*)	Increased protein synthesis rate at day 4 and decreased protein degradation rate at days 4 and 7 of culture development	(31)
	Recombinant human IGF-1 and IGF-2	Primary myocytes	Gilthead sea bream (*Sparus aurata*)	Stimulated cell proliferation (percentage of PCNA positive cells) by both IGF-1 and IGF-2 at day 4 of culture development	(32)
	Recombinant salmon/trout IGF-1	Primary myocytes	Rainbow trout (*Onchorynchus mykiss*)	Stimulated phosphorylation of Akt at Ser^473^, FoxO1 at Ser^319^ and FoxO4 at Ser^262^ at day 7 of culture Prevented the nuclear translocation of FoxO1	(33)
	Recombinant human IGF-1 and IGF-2	Primary myocytes	Gilthead sea bream (*Sparus aurata*)	Stimulated L-alanine and glucose uptake by IGF-1 and IGF-2 at days 4 and 9 Increased GLUT4 protein levels by IGF-1 and IGF-2 at day 9 Stimulated phosphorylation of Akt and MAPK proteins by IGF-1 and IGF-2 at day 4	(34)
	Recombinant human IGF-1	Primary myocytes	Rainbow trout (*Onchorynchus mykiss*)	Stimulated cell proliferation in a dose-dependent manner Increased gene expression of markers for early (*myf5* and *myod1*) and mid (*myogenin*) differentiation at day 7 of culture Reduced *mstn-1a* (day 7) and *mstn-1b* (day 3) mRNA levels and increased *mstn-2a* mRNA levels (days 3 and 7) Stimulated the processing of MSTN-2a mature transcript	(35)
	Recombinant human IGF-1	Primary myocytes	Rainbow trout (*Onchorynchus mykiss*)	Upregulated expression of the late marker of differentiation *mlc* at days 5, 10 and 14 Reduced *myod1* (day 5) and *myogenin* (days 5 and 10) mRNA levels Reduced *mstn-1a* and *mstn-1b* mRNA levels at days 5 and 10 and increased *mstn-2a* mRNA levels at days 5, 10 and 14 of culture	(29)
	Recombinant human IGF-1 and IGF-2	Primary myocytes	Gilthead sea bream (*Sparus aurata*)	IGF-1 upregulated markers of muscle differentiation (*mrf4* and *myogenin*), while IGF-2 upregulated markers of early muscle cell proliferation (*myod2* and *myf5*) Increased *igf-1* gene expression by IGF-2	(36)
	Overexpression of the zebrafish IGF-1 cDNA in skeletal muscle	Skeletal muscle	Transgenic crucian carp (*Carassius auratus*)	Decreased body weight Shift in the myofiber type toward a more oxidative slow muscle type IGF-1 signaling, aerobic metabolism, and protein degradation pathways were activated Higher oxygen consumption rates	(37)
	Recombinant gilthead sea bream IGF-1	Primary myoblasts	Pacu (*Piaractus mesopotamicus*)	Modulated IGF/PI3K signaling, amino acids metabolism and matrix organization High hybridization in *mmp14b/miR-338-5p* interaction Decreased gene expression of the muscle miRNA *miR-29b*, *mmp14b*, *fbxo25* and *tgfbr2* Increased gene expression of the muscle miRNA *miR-338-5p*	(38)
TGF-β	Inactivation of *TGFr*/overexpression *follistatin*	Muscle tissue	Rainbow trout (*Onchorynchus mykiss*)	TGF-β2, TGF-β3 and inhbA1 are involved in muscle growth or regeneration	(39–41)
	Morpholino *Smad4*	Cardiac and skeletal tissue	Zebrafish (*Danio rerio*)	TGF-β and Smad4 are essential for cardiac and skeletal development	(42)
IL-6	Muscle tissue		Rainbow trout (*Onchorynchus mykiss*)	No increase in *il-6* expression in muscle after exercise	(43)

### 2.1 Adiponectin

Adiponectin is a cytokine-like peptide produced primarily by adipose tissue in mammals, which plays a crucial role in lipid and glucose metabolism regulation. The first identification of adiponectin in fish was reported in zebrafish (44). Since then, this adipokine has been identified in other fish species, such as rainbow trout (*Oncorhynchus mykiss*), ayu (*Plecoglossus altivelis*), large yellow croaker (*Larimichthys crocea*) and common carp (*Cyprinus carpio*) (8, 45–47). The main regions of mammalian adiponectin have been analyzed in fish, and the globular domain, which is involved in receptor binding, is the most conserved one. However, although mammalian adiponectin can be found in monomeric or multimeric forms, until now there is no evidence on multimer formation in teleost species (reviewed by Ji et al. (8)).

Yet, the *adiponectin* gene might have been lost in some fish species throughout evolution since it was not found in some published genome databases. This is the case of Nile tilapia (*Oreochromis niloticus*), black carp (*Mylopharyngodon piceus*), Japanese puffer (*Takifugu rubripes*), medaka (*Oryzias latipes*), three-spined stickleback (*Gasterosteus aculeatus*) and green spotted puffer (*Tetraodon nigroviridis*) (8). On the other hand, there are two isoforms of *adiponectin* genes, namely *adipoqa* and *adipoqb*, in cyprinid fishes, including grass carp (*Ctenopharyngodon idella*) (48) and zebrafish (44). For perciform fishes, only one *adiponectin* gene has been reported in the literature.

The highest expression level of *adiponectin* in ayu is found in the adipose tissue, as reported in mammals (49). In zebrafish, rainbow trout, large yellow croaker, common carp, and grass carp, the expression in the muscle is higher than that in the adipose tissue (5, 8, 44, 45, 47, 48), but still remains to be evaluated whether this peptide could be also considered a myokine in these species. *adiponectin* is also expressed in other tissues, such as brain, liver, kidney or heart with variable levels depending on the species (8). In fish, there are almost no studies on adiponectin plasma levels (7), neither on peptide secretion, nor on peptide release into the circulation, which makes difficult to understand its role in inter-organ communication.

Regarding adiponectin receptors, and similarly to mammals, some fish species have two forms (AdipoR1 and AdipoR2), while zebrafish, grass carp and common carp have two isoforms of AdipoR1 besides AdipoR2 (44, 47, 48). The homologies with mammalian sequences are between 70 and 80%. AdipoR1 is mainly located in muscle and AdipoR2 in liver of humans or mice, but a wider distribution is observed in fish. In particular, in zebrafish and rainbow trout, they are widely distributed and can be considered as ubiquitous (5, 44). AdipoRs are also widespread in other teleosts, although the pattern of expression varies among species, suggesting this scenario possible inter-species differences in the function of adiponectin in the communication between tissues.

Concerning the factors regulating gene expression, insulin was able to stimulate *adiponectin*, and to decrease *adipor1* mRNA levels in rainbow trout cultured adipocytes (6) and *in vivo* in adipose tissue (5), in agreement with reports in various mammalian models (46, 50–52).

In mammals, adiponectin stimulates fatty acid oxidation, decreases plasma triglycerides and improves glucose metabolism by increasing insulin sensitivity (53, 54). Considering that many of its beneficial effects in mammals occur through the interaction with the skeletal muscle, it is not surprising that some physiological studies have focused on the role of adiponectin in fish muscle, specifically in trout cultured myocytes (5). In this *in vitro* model, no changes in glucose or fatty acids uptake were observed after a short period of incubation with adiponectin. Nevertheless, after 2 days of treatment, the reduced presence of fatty acids in the culture medium, and the increase in intracellular triglycerides, suggested an increase in fatty acid uptake. These effects were not accompanied by an increase in fatty acid transporters’ expression in trout, but in large yellow croaker myocytes, adiponectin upregulated the gene expression of the fatty acid transporters *cd36*, *fabp10* and *fabp11* (8).

Adiponectin also increased fatty acid oxidation in trout myocytes measured as CO_2_ production (5), one of the main actions of this adipokine in mammalian models, that helps to maintain lipid homeostasis and insulin sensitivity. Moreover, in trout myocytes, adiponectin activated the PI3K/AKT pathway but not the signaling through p44/42 mitogen-activated protein kinases (MAPK), similarly as it occurs in mouse myocytes (55). Indeed, in that study, wortmannin blocked the stimulatory effect of adiponectin demonstrating the specificity of the pathway. Activation of PI3K/AKT has been associated to increases in glucose and fatty acid uptake and beta-oxidation in mammals (56). The relevance of this molecular pathway in fatty acid oxidation in fish remains to be elucidated, but it appears that the regulation of muscle lipid metabolism by adiponectin and its mechanisms of action would be both conserved.

With regards to the effects of adiponectin regulating bone mass, controversial results were reported in mammals. From the available literature, it seems that adiponectin directly influences skeletal health. To identify the mechanisms that underlie the activity of adiponectin in bone, many studies *in vitro* and in mice models over-expressing or lacking adiponectin have been carried out. Although the *in vitro* data showed an osteogenic role of adiponectin, supported by pro-osteoblastic and anti-osteoclastic effects, data from transgenic mice or human studies failed to demonstrate unambiguously a beneficial effect of adiponectin on bone development (57). The inconsistency of the results may be partly due to the variation among experimental systems, the use of different structural forms and the complex nature of adiponectin signaling, which involves multiple direct and indirect mechanisms (58).

In fish, limited information exists on the role of adiponectin in bone. Zebrafish fed a high fat diet showed decreased plasma levels of adiponectin accompanied by a reduction of mineralized areas in scales and an alteration of growth tissue markers, such as a decrease of alkaline phosphatase and an increase in tartrate-resistant acid phosphatase activity respect to control fish, suggesting a positive effect of adiponectin on bone growth (7). Nevertheless, as far as we know, no other studies about the communication of adipose tissue and bone mediated by adiponectin have been addressed until now in fish.

Adiponectin has also local effects on the adipose tissue, stimulating glucose uptake and increasing lipid content in mammalian adipocytes (59). In fish, adiponectin increased glucose uptake in cultured rainbow trout adipocytes without modification of AKT or TOR phosphorylation (6), suggesting other signaling pathways involved. Nevertheless, due to the high levels of *adiponectin* expression in trout muscle, it is unknown whether an autocrine effect could exist *in vivo*, or whether adiponectin from muscle could contribute to regulate adipose tissue glucose metabolism in an endocrine or paracrine way. The future development of specific methods to measure circulating adiponectin levels and tissue secretion would help to better understand its role in fish.

Finally, it is remarkable that adiponectin and its receptors are modulated by nutritional status. For example, feed restriction affects their expression in a different way in muscle and adipose tissue in rainbow trout, Atlantic salmon (*Salmo salar*) and zebrafish, with also variations between species, underlining its key role in the communication between tissues (5, 44, 60). Nevertheless, going into detail about these changes is beyond the scope of this review.

Although the information regarding fish adiponectin is limited to very few species, taken together the reviewed results, a general conservation of structure and functions of adiponectin could be suggested at least bearing in mind its effects in muscle, bone, and adipose tissue metabolism. However, specific features in teleost adiponectin system should be considered to fully understand its physiological role.

### 2.2 Leptin

Leptin is a 16 kDa pleiotropic cytokine with an important role in food intake control and energy homeostasis in vertebrates. Fish leptin was first identified in 2005, when the *leptin* gene ortholog was cloned in pufferfish (61). Since then, leptin sequences were described in many fish species (reviewed by Blanco and Soengas (62)): common carp (63), zebrafish (64), medaka (65), Atlantic salmon (66), striped bass (*Morone saxatilis*) (67), yellow catfish, (*Pelteobagrus fulvidraco*) (68), mandarin fish (*Siniperca chuatsi*) (69), goldfish (*Carassius auratus*) (70), European and Japanese eel (*Anguilla anguilla* and *Anguilla japonica*, respectively) (71) and, more recently, in Northern snakehead (*Channa argus*) (72), among others.

Teleosts share very low primary sequence identity (~13-25%) with human *leptin* (73). A great number of fish species have two copies of this gene, named differently in various species (*lep-a/I/1* and *lep-b/II/2*), due to the whole-genome duplication (WGD) event that occurred during the evolution of the teleost lineage (73). For instance, two copies are found in tongue sole (*Cynoglossus semilaevis*), Nile tilapia, orange-spotted grouper (*Epinephelus coioides*), chub mackerel (*Scomber japonicus*), Northern snakehead, zebrafish and medaka (62). Both *leptin* sequences show variable identity and even duplicate *leptin* genes described within fish species may be very different in primary amino acid sequence conservation (e.g., zebrafish) (64). *lep-a/I/1* is the predominant form expressed in many of the teleost fish analyzed (74). On the contrary, other fish species (e.g., mandarin fish, pufferfish) retained only one *leptin* gene, but the existence of a second paralog cannot be discarded.

In mammals, leptin is an adipostat, is produced by, and circulates in proportion to the amount of white adipose tissue (75), but this scenario has not been confirmed yet in fish. In contrast to mammals, fish leptin shows differential distribution patterns, and is commonly highly expressed in the liver (62). Only in some species, the expression of *leptin* in adipose tissue is relatively high (76–78). Besides, its expression is found in some other tissues such as brain, gonads, kidney, gill, muscle and spleen, but the transcriptional patterns are very different between species (69, 79, 80). *leptin* paralogs also showed different transcriptional features within the same species, with *lepA* being more abundant in the liver whereas *lepB* in the gonads, thus indicating divergent roles (65, 76).

The development of different methods for measuring leptin in fish has resulted in abundant literature regarding plasma levels and their regulation, especially by nutritional status. In many fish species, and in contrast with mammals, circulating leptin was significantly increased after different periods of food deprivation, and subsequently decreased after refeeding in rainbow trout (68, 81, 82), or fine flounder *Paralichthys adspersus* (83). On the contrary, circulating leptin levels in other species were decreased after food deprivation (e.g., burbot, *Lota lota*) (84). Moreover, *leptin* expression in liver increased after refeeding in goldfish (70), common carp (63) or the cyprinid *Labeo rohita* (85). In addition, other studies revealed that leptin levels were not affected by fasting, but displayed peri-prandial changes in pacu (*Piaractus mesopotamicus*) (80). These findings suggested that leptin is clearly involved in energy homeostasis regulation but might exhibit different functional properties depending on the fish species. However, in all the species tested, leptin acted as an anorexigenic peptide; thus, increased circulating levels of leptin during fasting, reflected a possible strategy to avoid wasting energy foraging in those situations (62, 74).

Secretion studies are crucial to know which tissues might contribute to circulating leptin. Measurable levels of leptin A were found by homologous radioimmunoassay (81) in culture media from both, rainbow trout pre-adipocytes and mature adipocytes, with significantly higher levels in the latter (9). As far as we know, this study is unique demonstrating that leptin is produced and released by adipocytes and supporting that adipose tissue might contribute to circulating leptin levels in rainbow trout and that this can act as a hormone.

Nevertheless, as indicated in fish, *leptin* is highest expressed in liver. It is not surprising that leptin, as a regulator of energy expenditure, is controlled at least in part by glucose and key elements of the endocrine stress axis. In this sense, recently, Mankiewicz et al. (86), using tilapia (*Oreochromis mossambicus*) freshly isolated hepatocytes’ incubations and a homologous leptin A ELISA, showed that its secretion is modulated by high levels of glucose. In the same study, an *in vivo* injection of epinephrine stimulated a rapid rise in blood glucose, which was followed by a 4-fold increase in hepatic *lepA* mRNA and plasma levels. Interestingly, specific fatty acids could modulate fish leptin secretion since *leptin* mRNA expression was downregulated by saturated fatty acids, and upregulated by monounsaturated and long chain polyunsaturated fatty acids in rainbow trout liver slices (87).

Indeed, it would be very helpful to have more information on leptin secretion by hepatocytes and adipocytes and about its regulation especially in species where *leptin* is highly expressed in liver and adipose tissue. Furthermore, the co-culture of different types of fish cells, such as adipocytes-myocytes, may help to understand the interactions of their secretomes and the crosstalk between these tissues.

The physiological actions of leptin are mediated by a glycoprotein consisting of a single-membrane-spanning receptor (LepR), that belongs to the class-I cytokine receptor family (88). In fish, an orthologous gene for the mammalian *lepr* was identified in several species (62), typically present as a single ortholog; however, duplicate *lepr* paralogs have been identified in a few species, including Atlantic salmon (89), European eel (71) or rainbow trout (86). Furthermore, in some species more than one isoform is present, as it also occurs in mammals, generated by alternative mRNA splicing and/or proteolytic processing of the protein products (88). The mammalian long isoform, or *leprb*, is the only one with clearly demonstrated signaling capability (90). There is few information regarding the functionality of these isoforms, but the salmon LepR (long form) includes all functionally important domains conserved among vertebrate LepRs.

High expression of *lepr* is commonly found in the brain, pituitary, and gonads of many fish species; however, there are species-specific differences in its tissue distribution. In this sense, the muscle, head kidney, pituitary and pancreas are the major expressing tissues in Nile tilapia (78) and, the muscle, skin, gill, brain and eye in medaka (65). Nevertheless, the physiological role of these receptors is still unclear.

In vertebrates, adipocytes regulate energy metabolism of the whole body, including muscle and bone, indirectly through interaction of leptin with sophisticated brain circuits that maintain energy levels by affecting food intake and energy expenditure (91). In mammals, leptin increases sympathetic nervous system tone (92) and, through its interaction with the melanocortin system, activates thyrotropin-releasing hormone to increase thyroid hormone signaling and, thus, energy expenditure (93–95). As mentioned before, most of the studies of leptin administration in many fish species support an anorexigenic action and there is abundant literature regarding its role as inhibitor of food intake, which has been reviewed extensively (62, 73) and is out of the scope of this review. However, its role as a regulator of energy expenditure has been less explored in fish.

Regarding lipid homeostasis, autocrine effects of leptin on adipose tissue of rainbow trout have been suggested using recombinant trout leptin (96). Indeed, leptin increased lipolysis measured as glycerol release in mature freshly isolated adipocytes of this salmonid. Furthermore, leptin significantly suppressed the fatty acid transporter *fatp1* expression, suggesting a decrease in fatty acid uptake and storage, but did not affect the expression of any of the lipogenesis or β-oxidation-related genes studied (9). More recently, Basto-Silva et al. (11), reported that leptin inhibits lipid accumulation, significantly reducing the peroxisome proliferator-activated receptor gamma (*pparγ*) and fatty acid transporter *cd36* gene expression, both in early differentiating and mature adipocytes of gilthead sea bream (*Sparus aurata*).

Some other findings come from studies with *lepr* mutants. Indeed, hyperphagia in *lepr*-deficient medaka led to a higher growth rate at the post-juvenile stage, but not in adults, although adult *lepr* mutants possessed large depots of visceral fat, unlike the wild type fish (97). On the other hand, *lepr* knockout zebrafish did not develop obesity (98), which is in marked contrast to mammals, particularly mice (ob and db models) and barely affected metabolism and energy allocation (99). *lepr* knockout trout exhibited a hyperphagic phenotype, and increased energy stores in muscle as observed in mammalian models (100). Recently, it was reported that leptin induces muscle wasting in a zebrafish *kras*-driven hepatocellular carcinoma model (10). By using *lepr* knockout fish, it was found that these animals had a higher survival rate and significantly lower muscle-wasting level after tumor induction than the tumor-induced fish in the wild-type background. These results, besides having interest in cancer cachexia, also demonstrated direct effects of leptin in fish muscle physiology.

In mammals, leptin has intense effects on skeletal muscle fatty acid metabolism, resulting in an increase in the capacity of this tissue to oxidize fatty acids and reduce triacylglycerides stores. Therefore, the development of leptin resistance in skeletal muscle, characteristic of obesity, may lead to insulin resistance in that tissue by allowing the accumulation of intramuscular lipids and disruption of the insulin signaling pathway (101). This situation can be ameliorated by different lifestyle factors such as aerobic training and diet, establishing a homeostatic crosstalk. Although not being an obesity fish model, studies in energetically divergent rainbow trout lines selected for low (lean line, LL) and high (fat line, FL) muscle adiposity, revealed impaired central leptin signaling system in the FL fish, probably linked to high muscle fat, since no differences in appetite regulation and feed intake between the two rainbow trout lines were found (102).

Some studies suggested indirectly the regulation of muscle metabolism by leptin in fish. The increase in circulating leptin under food deprivation might trigger the activation of the AMP-activated protein kinase (AMPK) in the skeletal muscle of fine flounder and contribute to the negative effects on anabolic processes (83). Another indirect evidence of leptin actions on fish muscle came from studies on *lepr* expression, which is modulated by nutritional status. The two *lepr* paralogs identified in rainbow trout are differentially expressed across tissues under catabolic conditions, showing that during fasting, leptin is likely acting to promote energy mobilization in the muscle through *lepra1* and in the liver through *lepra2* (86).

Clearly, more studies are required to learn about leptin crosstalk. The use of fish models in which primary culture of myogenic cells is well established (27, 30) may provide further insights for understanding the mechanisms by which leptin regulates metabolism and growth in fish skeletal muscle.

Regarding bone as a target tissue of leptin, different studies with mammalian models have shown that it plays multiple crucial roles in skeletal growth and metabolism through both, central and peripheral pathways, and that it might be involved in many human bone diseases (103). In humans, leptin is likely to exert a positive effect on bone mass, and contribute to the balance between adiposity and bone density (104). Interestingly, administration of a high fat diet alters energy metabolism generating an osteoporosis-like phenotype in adult zebrafish scales (7), but no information regarding the possible role of leptin regulating these processes has been reported to date. In this sense, it would be very interesting to study the role of leptin regulating the plasticity of cells derived from gilthead sea bream vertebra bone, which can be differentiated into both, osteoblast and adipocyte phenotypes (105, 106), as this will shed more light into the possible role of leptin as a mediator of the communication between adipose tissue and bone in fish.

### 2.3 Resistin

Resistin is a peptide that was first identified as a factor produced exclusively by adipocytes (107). Indeed, in mice, *resistin* mRNA expression is abundant only in adipose tissue (107) while in humans, it is expressed in white adipose tissue but also in other tissues. In fact, peripheral blood mononuclear cells, macrophages, and bone marrow cells are the primary source of circulating human resistin (108). Resistin is widely found in other species and, for example, it is highly expressed in the lung in porcine, goat and yak (12).

The increase in plasma resistin concentration impairs insulin sensitivity and decreases glucose tolerance in mice, and plays a significant role in human obesity-induced insulin resistance (109). It also shows pleiotropic effects and could be involved in physiological and pathological processes of bone/rheumatological disorders. The resistin receptor is unknown but several proteins could do such function: toll-like receptor 4, decorin, orphan receptor-1, insulin-like growth factor receptor, and adenylyl cyclase-associated protein 1 (110, 111).

In 2015, Hu and co-workers analyzed available genome sequences of the *resistin-like* gene family of diverse vertebrates (112). Genes encoding resistin-like peptides were found in Actinistia (coelacanth, a lobe-finned fish), but not in fish from the Actinopterygii, Chondrichthyes, or Agnatha. More recently, a *resistin* full-length cDNA has been obtained, with an open reading frame encoding 106 amino acids in Siberian sturgeon (*Acipenser baerii*) (12), showing low identity with other vertebrates, except when compared with the sterlet (*Acipenser ruthenus*). However, the resistin C-terminal is well conserved among species, suggesting that this region may be important to its physiological roles.

Gene expression of *resistin* was very high in the liver of Siberian sturgeon in comparison with other tissues, as described previously also for *leptin*. Besides adipose tissue, the liver is an important organ of lipid accumulation in some species, and it is essential in metabolic homeostasis, suggesting that resistin, as other classical adipokines, may have a role in metabolism regulation. In fact, in Siberian sturgeon, *resistin* mRNA expression in the liver decreased after fasting and increased sharply after refeeding, confirming it is affected by nutritional status (12) supporting this hypothesis. Nonetheless, data is limited to this study; consequently, further studies are needed to investigate the role of resistin as a hormone involved in tissue crosstalk in fish.

### 2.4 Tumor necrosis factor

TNF is a cytokine belonging to the “TNF ligand superfamily” (113, 114) that plays crucial roles in regulating immune functions and metabolism in vertebrates. In mammals has two isoforms, TNFα and TNFβ, while in fish there is only one more similar in structure and organization to TNFα (115). Only a single copy of *tfnα* gene was found in mammals, otherwise, various copies of the same gene were found in fish, the number of which depends on the species (116–122). TNFα was first identified in the Japanese flounder, *Paralichthys olivaceus* (123), and posteriorly in zebrafish, rainbow trout, gilthead sea bream and common carp (120, 124, 125). TNF family signature is well conserved among mammals and fish (119, 126).

TNFα was first described as a cytokine secreted by macrophages regulating inflammation, apoptosis, cell proliferation and stimulation of the immune system (115).. TNFα is also secreted by adipose tissue or skeletal muscle (113, 127), and it is classically included in the list of mammalian adipokines. It was in 1999 when Bulló-Bonet et al. (128) suggested that TNFα can be produced by human adipocytes themselves and, more recently, *tnfα* expression has also been detected in fish cultured adipocytes (6).

TNFα receptors, TNFR1 and TNFR2, have been described for several fish species (129). The functions of TNFR1 are, among others, related to apoptosis or cellular death while TNFR2 is involved in the promotion of cell survival (129). *tnfr1* is expressed in many tissues, but *tnfr2* is more tissue specific, limited to immune, endothelial, microglia and nerve cells (129).

Either the mature adipocytes or the stromal vascular fraction of adipose tissue, which includes pre-adipocytes mixed with macrophages, can act as endocrine cells secreting adipokines including TNFα. In mammals, obese individuals produce more TNFα, in part due to the high number of infiltrated macrophages in the adipose tissue (6, 130, 131). TNFα has a clear paracrine and/or autocrine role within adipose tissue (17). In mammals and fish, TFNα promotes adipocyte lipolysis, modulates lipogenesis, and glucose transport and inhibits pre-adipocytes proliferation (129, 132). It can also interact with other cytokines and vice versa (115). In fish, as in mammals, besides modulating fat metabolism, it has been proved that TFNα can act as a pro-inflammatory, apoptotic or organ regenerator regulator (6, 133–136). In almost all fish studies conducted and described in this review, mammalian recombinant TNFs have been used (5, 13, 137), although few studies have reported the use of recombinant trout TNF1 and TNF2 in muscle or other cell models (16, 129, 138).

Most of the studies regarding the effects of TNFα as a crosstalk mediator in fish have been limited to its autocrine effect in adipose tissue. This cytokine induced lipolysis in rainbow trout (13), gilthead sea bream (18, 19) or large yellow croaker (17) in experiments conducted both *in vitro* and *in vivo*. In mammalian models, TNFα lipolytic action is usually mediated by modulation of hormone-sensitive lipase (HSL) and adipose triglyceride lipase (ATGL). Silencing of *atgl* expression in 3T3-L1 pre-adipocytes almost completely abolished TNFα-induced glycerol release, while TNFα-induced lipolysis under the same conditions was only partially decreased upon reduction of *hsl* expression (139). In contrast with these observations, TNFα decreased the expression of the key lipolytic enzyme *atgl* in large yellow croaker pre-adipocytes (17) although other mammalian studies showed a similar action (140–142). The expression level of *atgl* was increased in liver and muscle by lipopolysaccharide (LPS)-induced TNFα in blunt snout bream, but not in adipose tissue (143). Altogether, these data suggest that TNFα might modulate adipose tissue lipolysis at several levels and with differences between fish species. These variations might be explained in terms of differences in the doses and origin of the peptides used in the experiments.

Regarding mechanisms of action, TNFα can activate MAPK in human adipocytes, leading to a lipolysis increase though ERK1/2 and p38 kinase activation (144, 145). Accordingly, Albalat et al. (13) demonstrated that these protein kinases are partially involved in the lipolytic effects of human TNFα in rainbow trout adipocytes. On the other hand, TNFα increased PPARα during activation of adipocyte lipolysis in large yellow croaker, in agreement with the role of this transcription factor in fatty acid catabolism (17, 146, 147). The relevance of PPARγ is not so clear as TNFα downregulated *pparγ* expression, promoting lipolysis in gilthead sea bream adipocytes (19) as in mammals (148), but during TNFα-induced lipolysis, *pparγ* expression levels increased in large yellow croaker (17). With regards to other possible transcription factors involved, liver X receptor (*lxr*) was upregulated by TNFα in rainbow trout isolated adipocytes, in parallel to the pro-lipolytic actions of this factor (14). Nevertheless, this mechanism of action is not so clear in TNFα-induced lipolysis in gilthead sea bream isolated adipocytes (19), highlighting again the complexity of the signaling pathways of TNFα activating adipocyte lipolysis in fish.

Furthermore, TNFα can cause a decrease in adiposity by other mechanisms besides inducing lipolysis. In order to decrease the amount of triglycerides in adipocytes, TNFα minimizes fatty acid uptake reducing substrate availability, but also inhibiting the esterification of free fatty acids in mammals (132) or decreasing the lipogenic enzyme fatty acid synthase (*fas*) expression (149). Among fish studies, TNFα did not change the expression of *fas*, *pparα* or *pparγ* in large yellow croaker pre-adipocytes but decreased the expression of lipoprotein lipase (*lpl*) (17), in agreement with its role reducing fatty acid uptake. Supporting this fact, in rainbow trout, TNFα induced by LPS administration, decreased *fatp1* mRNA expression in adipose tissue, as well as in isolated adipocytes (5).

Moreover, in mammals it is proved that TNFα induces insulin-resistance (12, 150), by impairing insulin-stimulated glucose uptake through the inhibition of insulin signaling at the insulin receptor substrate level (151, 152), but on the other hand, TNFα increases basal glucose uptake in adipose tissue (153). From studies in rainbow trout adipocytes, it was concluded that stimulation of basal glucose uptake by TNFα seems to be conserved from fish to mammals. Meanwhile, the interactions between insulin and TNFα are ambiguous in fish, namely: although an inhibitory effect compared to insulin treatment was observed on AKT and TOR phosphorylation when TNFα was added together with insulin in rainbow trout adipocytes, it was not reflected on glucose uptake (6). On the other hand, recombinant trout TNFα directly stimulated glucose uptake in rainbow trout myoblasts and myotubes providing evidence for a potential regulatory role of TNFα in skeletal muscle metabolism (16).

Concerning adipogenesis, a negative effect of TNFα has been well documented in several *in vitro* models (12, 154, 155), including fish pre-adipocytes and adipocytes (6, 15). In mammals, TNFα is both an inhibitor of adipocyte differentiation and a suppressor of some early stage genes responsible of pre-adipocyte conversion (156); although in rats or mice, low concentrations of TNFα have a promoting effect on adipocyte proliferation (155, 157). In large yellow croaker, high concentrations of TNFα inhibited adipocyte proliferation but low concentrations had no effect (17). Like in mammals, this cytokine slightly enhanced rainbow trout adipocyte proliferation, only at a low dose, and inhibited cell differentiation, as indicated by a decrease in glycerol-3-phosphate dehydrogenase activity (15). Additionally, *lpl*, proved to be an early marker of adipocyte differentiation, together with *pparγ*, were transcriptionally downregulated by TNFα in fish as in mammals (15, 17). Furthermore, in the primary rainbow trout adipocytes culture, incubation with TNFα downregulated the expression of *fatp1* (5), which can be understood not only as a decrease in cell differentiation but also as a contribution to reduce fatty acid uptake and thus, the lipid content of the adipocyte. Moreover, TNFα could cause a decrease in adiposity in mammals inducing adipocyte death (132, 158, 159), but as far as we know, there is no data available on the cytotoxic effect of TNFα in fish adipose tissue. Overall, differences in the response of adipocyte cells to TNFα in fish seem to be related to dosage and species specificity as it occurs in some mammalian models as well.

Interactions between TNFα and other adipokines (i.e., adiponectin) seem to have specific features in fish. Indeed, it has been reported that adiponectin suppresses the expression and release of TNFα from human and 3T3-L1 adipocytes (160, 161). In rainbow trout adipocytes, TNFα increased the mRNA levels of *adipor1* but neither *adiponectin* nor TNFα modulated each other gene’s expression, although TNFα inhibited the insulin-induced increase in *adiponectin* mRNA levels (6). Therefore, the reciprocal suppressive effect of both adipokines previously reported in mammals is not clearly present in fish. When tested *in vivo*, the absence of a mammalian-type regulation between adiponectin and TNFα was confirmed, since TNFα injections failed to regulate the expression of the adiponectin system in rainbow trout adipose tissue (5).

To sum up, TNFα functions seem to be conserved between fish and mammals, it is secreted by fish adipocytes and could act in the adipose tissue in an autocrine way, and in other tissues including skeletal muscle. Therefore, it is likely that a crosstalk exists among all the tissues in which TNFα is involved, but further studies are needed to definitively prove that.

### 2.5 Other adipokines

Another regulatory peptide produced by adipocytes to be considered is apelin. In mammals, apelin and its receptor, that belongs to the type 1 angiotensin G protein-coupled receptor family (162), are expressed in adipose tissue but also widely distributed in other peripheral tissues in goldfish (162), Ya-fish (*Schizothorax prenanti*) (163), and others (164). Apelin has multiple roles, according to the numerous locations where it is found. These include the regulation of cardiovascular functions, metabolism, feeding behavior and energy expenditure. In adipose tissue and muscle, apelin regulates AMPK and promotes glucose utilization. It was hypothesized that apelin contributes to obesity disorders by developing adipose tissue mass *via* angiogenesis or pre-adipocytes proliferation. Nevertheless, contradictory results among diverse studies in mammals have been described and the precise role of apelin needs further investigation (162, 163, 165, 166). In fish, very little is known regarding apelin. Apparently, the only role of apelin demonstrated in fish was as orexigenic factor (162, 163). Nonetheless, other studies have hypothesized that apelin might be involved in the regulation of many physiological functions in fish due to its expression in various tissues, as in mammals.

Concerning other adipokines, adipsin and visfatin are two recognized ones found in mammals and fish. Adipsin has been described in olive flounder (167) and visfatin in silver Prussian carp (*Carassius auratus gibelio*) (168). Both were identified in Atlantic salmon and expressed in differentiating adipocytes derived from the adipose-derived stromo-vascular fraction (169). These studies agreed with mammalian literature showing that, apart from being expressed in different tissues, they are found mainly in adipose tissue. In mammalian models, those cytokines are implicated in immunological and inflammatory functions and, could be also involved in the control of lipid metabolism and adipogenesis. The basis established in the literature about those two adipokines could help discovering in the future the true roles, pathways, and cross talking in which adipsin and visfatin are involved in fish.

## 3 Lipokines

Studies of mammalian adipose tissue secretome focused at first on adipokines (leptin and adiponectin, mainly); however, adipose-derived lipids or lipokines emerged as new endocrine factors (170). Provided by intracellular pathways of fatty acid metabolism, circulating in blood plasma and acting as mediators between adipose and non-adipose tissues (pancreas, liver or muscle), lipokines appeared as regulators of metabolic homeostasis, stress or inflammation (171). Among these lipokines, lysophosphatidic acid, palmitoleate, fatty acyl esters of hydroxy fatty acids, oxylipins and N-acyl amino acids, can act as autocrine or paracrine signals (114). Some lipokines increase fatty acids uptake in skeletal muscle (172), whereas others secreted by myocytes and stimulated through exercise (i.e., β-aminoisobutyric acid (173);), act upon white adipocytes and hepatocytes. Interestingly, lipidome atlases now appear to characterize adipose tissues: subcutaneous *versus* visceral depots (174).

On fish so far, few lipidome data are available in relation to crosstalk between organs, maybe due to a key fat storage in liver in some species, or only the existence of white fat adipose tissue, whereas in mammals, brown adipose tissue is involved as well (175, 176). To our knowledge, lipidome reports rather appeared in relation to fish environment or diet, focusing more on lipid metabolism than on lipokines (177, 178). For instance, Dreier et al. (177) highlighted the impact of environmental factors on lipid metabolism referring to studies on largemouth bass (*Micropterus salmoides*), zebrafish embryos or adults, rainbow trout juveniles or Mediterranean cyprinidae. Therein, relationships between pollution and changes in transcripts related to lipid metabolism were evidenced, as well as chemical exposure and fish lipid composition, or polluted water and phosphatidyl-cholines/-ethanolamines alterations within skeletal muscle. Among changes, the cholesterol pathway appeared as the one exhibiting the highest number of altered metabolites in the study on zebrafish embryos, whereas limited phospholipid lipase activities affected brain tissues, and neurobehavioral responses, in the report on young trout. On the other side, Jin et al. (178) reported how diet composition affects the lipid uptake (gut), processing (liver) and deposition (muscle) in Atlantic salmon in freshwater and seawater stages.

## 4 Myokines

**Figure 2:**
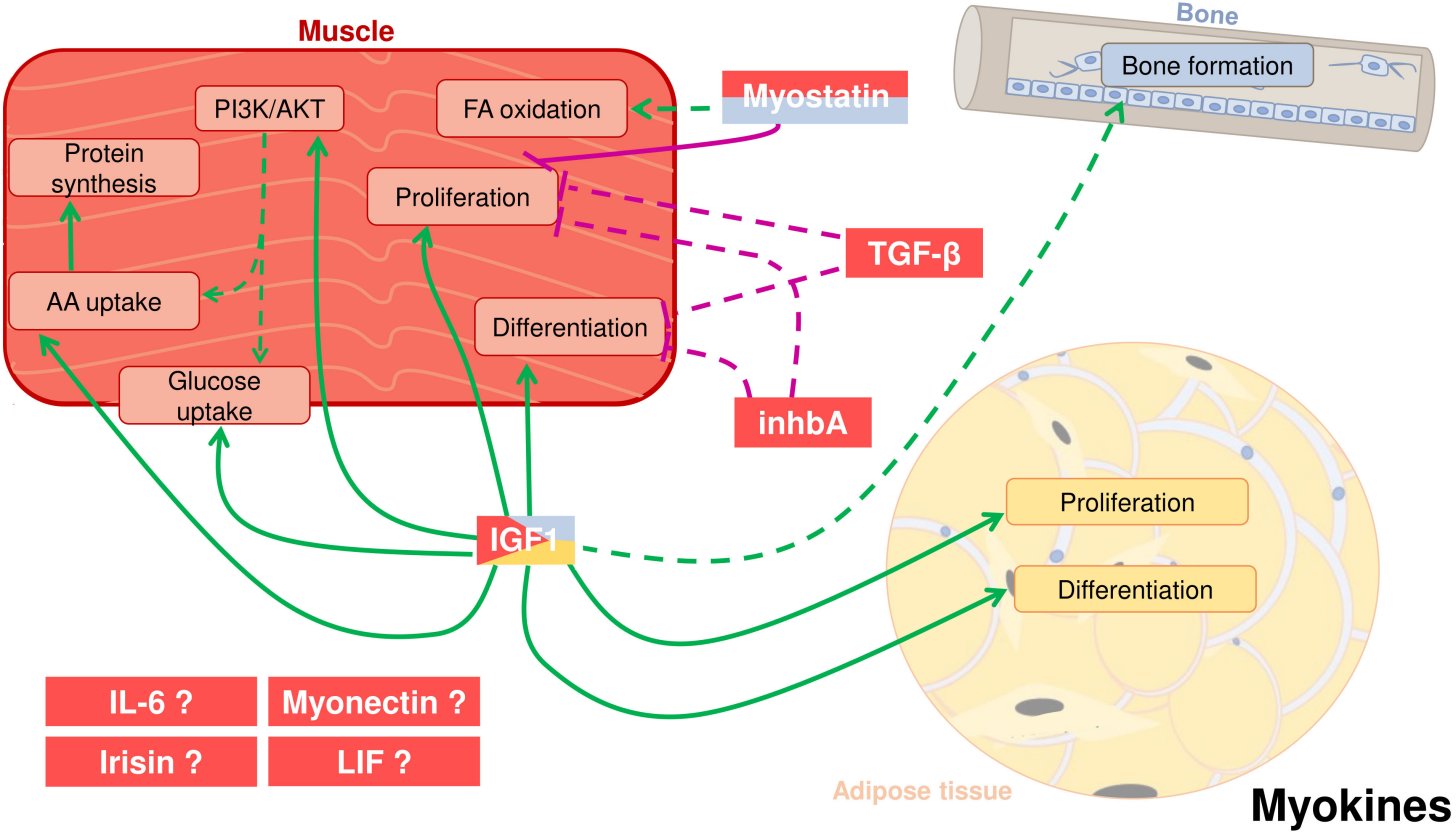
Crosstalk between adipose tissue, muscle, and bone in fish through cytokines secreted by the skeletal muscle, also known as myokines. Myostatin (MSTN) can be secreted by muscle but also from the bone. It has a potential positive effect on fatty acid (FA) oxidation and a proved negative effect on myocyte proliferation. Other two myokines most likely have autocrine roles: Transforming growth factor-β (TGF-β) and inhibin A (inhbA) inhibit cell proliferation and differentiation in muscle. Insulin-like growth factor 1 (IGF-1), produced mostly by muscle but also by other tissues like adipose and bone, enhances in muscle cell proliferation and differentiation, activation of the PI3K/AKT pathway, glucose and amino acid (AA) uptake and protein synthesis. IGF-1 also induces proliferation and differentiation of adipose cells. Moreover, it is also hypothesized that IGF-1 has an influence on bone formation. Other potential myokines are interleukin 6 (IL-6), irisin, myonectin, and leukemia inhibitory factor (LIF). Positive proved effects: green solid line; Positive suggested effects; green dotted line; Negative proved effects: purple solid line; Negative suggested effects: purple dotted line.

Skeletal muscle constitutes the largest part of the body weight, particularly in fish, where it accounts for up to 60-70% and is therefore the largest organ. Since the beginning of the 21^st^ century, it is known that skeletal muscle secretes proteins called myokines, which influence the function of other tissues and organs (4, 179) through autocrine, paracrine and endocrine signaling. Although several hundreds of myokines have been identified in mammals from decades, few are known in fish. These myokines and their reported effects in the muscle itself, bone and adipose tissue in fish are summarized in **Figure 2** and **Table 1**.

### 4.1 The transforming growth factors family members

The TFG beta (TGF-β) superfamily includes numerous structurally related growth factors known to regulate proliferation and differentiation of many cell types. These growth factors are classified into three subfamilies: TGF-β (including myostatin, MSTN), bone morphogenetic proteins and activin/inhibin.

### 4.2 Myostatin

The most famous and studied myokine is MSTN, also known as growth differentiation factor 8 (GDF-8) belonging to the TGF-β superfamily. MSTN is synthesized as a precursor protein containing a signal peptide, a large prodomain, and the bioactive peptide. After two cleavages, the mature MSTN dimer functions through binding to the activin receptor IIB that leads to the phosphorylation of the transcription factor Smad3 (180). Identified in 1997 in mouse by McPherron et al. (181), *mstn* is nearly expressed exclusively in skeletal muscle although it is also detectable in cardiac muscle and adipose tissue. The models of *mstn* knockout mouse or the natural null allele lead to the “double-muscle” phenotype (181, 182), being MSTN considered as the strongest endogenous inhibitor of muscle growth. Interestingly, the *mstn* knockout mouse beside the muscle phenotype, also exhibits decreased fat mass. Several works have shown that inactivation of MSTN increases the expression of key thermogenic genes (e.g., *ucp1*, *prdm16*) and brown adipose-related markers in white adipose tissues, leading to increased energy expenditure and loss of adipose mass (183, 184).

In fish, *mstn* genes were identified in numerous species revealing the presence of up to four paralogs coding for a well-conserved mature protein (~90% identity between mammals and fish) (reviewed by Gabillard et al. (185)). Fish *mstn* expression is not restricted to muscle but rather has a widespread pattern including white and red muscle, brain, liver, ovary… (20, 186–188). In addition, the expression of *mstn* paralogs is differently regulated according to tissues and physiological situation. In rainbow trout, *mstn-1a* is expressed in nearly all tissues whereas *mstn-1b* and *mstn-2a* are mainly expressed in brain, red and white muscle. In zebrafish and tilapia, *mstn-1* is strongly expressed in brain and muscle whereas *mstn-2* is mainly expressed in brain (185). Moreover, a fasting period upregulated *mstn-1* but not *mstn-2* expression in muscle and liver of barramundi (*Lates calcarifer*) but decreased *mstn-1* expression in brain and gills (20). On the contrary, in zebrafish, no clear effect of fasting was observed for both *mstn* orthologs in muscle (189). Concerning the receptor, expression of activin receptor type IIB has been found in numerous tissues in salmon, goldfish, and zebrafish, suggesting that MSTN could have an effect in several fish tissues including the muscle, hence acting in an autocrine manner (35).

To decipher the mechanism of action of MSTN in fish muscle, several studies used cultured myoblasts. With appropriate proliferation and differentiation media, it was shown that human MSTN inhibited trout myoblast proliferation (21) and induced myotube atrophy (22). In contrast, MSTN had no effect on myoblast differentiation because it did not decrease MyoD and Myogenin protein levels (21) as in mammals (190). Another study (35) observed that myoblasts cultured in low serum concentration (2%) expressed higher levels of myogenic markers (*myogenin*, *myod*) in the presence of MSTN, suggesting an enhancement of differentiation. Nevertheless, low serum concentration and addition of MSTN for a long time, strongly inhibited cell proliferation and consequently promoted differentiation in a non-specific manner as suggested by the authors themselves.

To understand the function of MSTN better, several authors inactivated the gene and observed the muscle phenotype. In medaka, a null-mutation of the unique *mstn* gene induced a 30% increase of muscle mass 16 weeks post-hatching (23), associated to fiber hyperplasia during the first 5 weeks post-hatching and fiber hypertrophy at 16 weeks of age. In the last few years, several papers have reported an increase of muscle mass following *mstn* disruption in carp (191), blunt snout bream (192), olive flounder (193) and channel catfish (194), showing in this case a consistent response between species. Unfortunately, the consequences of *mstn* null mutation on other tissues were not studied in these works. TALEN-induced inactivation of *mstn* gene in medaka observed an increase of muscle mass (25%) but also an alteration of the immune system (24). In zebrafish, inactivation of *mstnb* but not *mstna* lead to an increase of muscle mass after 80 days post-fertilization associated to muscle hyperplasia (25, 150). Interestingly, the authors reported an increase of fat accumulation in muscle of *mstnb* -/- zebrafish and showed evidences for a transition of energy supply from an amino acid- to a lipid-dependent source (150). Recently, CRISPR/Cas9 inactivation of *mstn* in loach (*Misgurnus anguillicaudatus*) led to an increase of fibers number but also accumulation of lipids, especially in red muscle (195).

Regarding the impacts on bone metabolism, *mstn* null mice displayed in some studies, besides muscle hypertrophy, a significant increase in bone mineral density, which provides direct evidence of muscle to bone biochemical crosstalk (196, 197). Some evidence exists that inhibition of MSTN action increases bone formation in mice, suggesting that it exerts a negative effect on osteoblasts differentiation. In contrast, MSTN acts as a positive regulator of osteoclasts, increasing bone resorption (198). Hence, fish *in vitro* studies using osteoblast cultures (105) would be very advantageous to study the possible communication of MSTN with fish skeletal bone.

Together, these results suggest that MSTN could have a role in non-muscle tissues, but it is unknown whether these effects result from crosstalk between tissues or simply from a wide expression of MSTN, for example in adipose tissue or immune system organs. In fact, bone-derived MSTN has been also recently suggested to control muscle development in gilthead sea bream in two *in vivo* models, one of fasting and refeeding and another one of muscle regeneration after an injury, since *mstn2* expression in bone was in all cases modulated, as proposed by the authors, to coordinate musculoskeletal growth (199, 200).

### 4.3 Other TGF-β family members

Whereas the function of MSTN is well studied in fish, data about the potential role of other TGF-β members on the crosstalk between adipose, muscle and bone tissues are very scarce. In trout, four *tgf-β* orthologs *(tgf-β1*, *tgf-β2*, *tgf-β3*, *tgf-β6*), three paralogs of *tgf-β1*, (*tgf-β1a*, *tgf-β1b* and *tgf-β1c*), and four *inhibin βA (inhba)* paralogs have been identified (201), expressed in red and white muscle and adipose tissue. It is noteworthy that *tgf-β6*, which is not present in tetrapods, is highly expressed in white muscle of gilthead sea bream (202) but not in trout (201). In addition, *tgf-β6* expression was downregulated during a refeeding period following fasting in gilthead sea bream muscle (202). Under a comparable experimental condition in trout, *tgf-β1a* and *tgf-β2* were quickly downregulated during refeeding whereas *tgf-β3* increased up to 7 days post-refeeding (201). In contrast, the muscle expression of *inhba1* but no other *inhba*, sharply dropped during refeeding being even undetectable in some fish samples. *inhba* was downregulated during *in vitro* differentiation of myogenic cells, and upregulated by IGF-1 (39). Similarly, *inhba* knockout mice exhibited enhancement of muscle development, showing a conserved role for this gene in growth regulation (203). Furthermore, Smad4, the central intracellular mediator of TGF-β signaling, has been shown to be essential for cardiac and skeletal development of zebrafish (42).

Moreover, TGF-β members and inhibin/activin bind to the activin type II receptor (AcvR) and overexpression of a dominant negative form of this receptor induced a strong development of trout muscle (40). In zebrafish, inactivation of both activin receptors (acvr2aa *and* acvr2ba) induced a strong increase of muscle mass due to fiber hypertrophy (204). Similarly, overexpression of *follistatin*, an antagonist of inhibin/activin and MSTN, specifically increased muscle growth (41). Together, despite the complexity of the TGFs superfamily reported in fish, the data suggest that some TGF-β members and inhbA could be inhibitors of muscle growth or regeneration in trout, even though these results need to be confirmed in other fish species.

### 4.4 Insulin-like growth factors

The growth factors IGF-1 and IGF-2 are polypeptides of 67-70 amino acids identified in mammals and fish 50 years ago, and well conserved across evolution (~70% amino acid identity between mammals and fish) (205). These growth factors are mainly produced and secreted from the liver, which accounts for 80% of the circulating IGF-1 (206), the peptide that exerts the major endocrine control of tissues growth. Nevertheless, *igfs* are expressed in almost all tissues, stimulating in an autocrine/paracrine manner, the proliferation and differentiation of cells. IGFs bind to a tyrosine kinase receptor, the IGF type 1 receptor (IGF1R) that mediates almost all the actions of IGF-1 and IGF-2 and has a ubiquitous expression. Another kind of receptor, the IGF type 2 receptor, exhibits greater affinity for IGF-2 than for IGF-1, but does not activate signaling pathways. In contrast, the binding of IGF-2 to its own receptor results in the degradation of the ligand, thus participating to the regulation of its activity. To define the effects of IGF-1 produced by muscle fibers, a transgenic construct was generated in which expression of a human *igf-1* was driven by the avian skeletal *α-actin* gene (207). IGF-1 concentration in the serum was similar in wild type and transgenic mice, but the latter developed skeletal muscle hypertrophy and male mice had less adipose tissue (208). The IGFs produced by skeletal muscle were considered myokines and modulated by several hormonal and environmental factors to regulate muscle homeostasis but also bone and adipose tissues.

Regarding endocrine or possible autocrine effects, in fish cultured myoblasts, IGFs stimulate cell proliferation (27, 30, 32), amino acid and glucose uptake, protein synthesis (22, 31, 33, 34), and cell differentiation (28, 35, 35, 38) contributing to muscle growth. In addition, growth hormone (GH) and IGF-1 stimulate the expression of *igf-1* in myoblasts of gilthead sea bream (209) and Atlantic salmon (29) supporting the promotion of autocrine functions.

From several decades, the effect of nutritional status on *igfs* expression has been studied in numerous fish species. For instance, food starvation results in suppressed growth associated with a decrease of liver *igf-1* mRNA and circulating IGF-1 in trout (36), eel (210), salmon (211), barramundi (212), catfish (213) and tilapia (214). In muscle, food restriction decreases *igf-1* but not *igf-2* expression in trout (36, 215, 216) and tilapia (217, 218) showing that muscular expression of *igf-2* is not related to muscle growth. According to these results, amino acids supplementation stimulated the expression of *igf-1* and *igf-2* in myoblasts/myotubes of salmon in culture (29). Another environmental parameter that influences expression of *igfs* is water temperature. Indeed, in juvenile trout, expression of *igf-1* in muscle is higher at 16°C than 8°C (219) whereas no significant effect has been observed for *igf-2* expression. Nevertheless, the induction of *igf-1* expression in muscle was rather due to a temperature-induced increase of food intake than to the temperature itself (219).

Exercise in fish often induces better growth rate, especially in pelagic fish. Recently, it has been reported that gilthead sea bream under sustained swimming expressed more *igf-1* in white muscle (220). More precisely, it was reported that the splice variant *igf-1c* was upregulated whereas no effect was observed for *igf-1a* and *igf-1b*. This effect was found in the anterior and the caudal part of the myotome, whereas *igf-2* expression was upregulated only in the caudal part, where the muscle is heavily loaded during swimming. In zebrafish, exercise stimulated muscle growth without stimulation of IGF-1 (221) highlighting potential differences between fish species. These differences could be attributed not only to fish species characteristics but also to the type of exercise (intensity and duration). Unfortunately, in those studies bone growth was not analyzed to enlighten a possible role of muscle IGF-1 acting locally on skeletal bone development/metabolism. To our knowledge, only one paper in fish is available showing *in vivo* evidence of the functional role of IGF-1 produced by muscle in an autocrine or paracrine way. These data showed that transgenic carp overexpressing *igf-1* in the white muscle exhibited lower growth and a fast-to-slow fiber switch associated to upregulation of the muscle genes involved in lipid metabolism (222). The authors suggested that the paradoxical lower growth rate reported could be related to deep changes in metabolism that would affect the growth of muscle fibers.

Notwithstanding, despite numerous data on IGFs actions and regulations, it remains unclear whether IGFs produced by the muscle have a function on the development of adipose tissue or bone as it occurs in mammals. In this sense, during bone repair, muscle-derived IGF-1 may signal to the osteoprogenitor cells in the periosteum to increase bone formation in a paracrine way (198). Supporting this hypothesis, increased proliferation was reported in response to IGF-1 in an *in vitro* model of bone-derived cells from gilthead sea bream (105). However, if this IGF-1 *in vivo* comes from the muscle or acts as an osteokine in an autocrine manner still must be deciphered.

Furthermore, we could also consider IGF-1 as an adipokine, as it is expressed together with its receptor in adipose tissue as in many other tissues in mammals and fish (43). Some studies have addressed this, and the mitogenic effect of IGF-1 in the adipose tissue has been confirmed. In this sense, IGF-1 increased proliferation of rainbow trout (15) and gilthead sea bream (37) pre-adipocytes and, it also stimulated differentiation in the early stages of gilthead sea bream cells in culture (37). Again, we cannot categorize what type of communication is really happening *in vivo* considering that this IGF-1 could be coming from the adipose tissue and act in an autocrine way, but also from contiguous tissues such as muscle or from the plasma being IGF-1 produced in the liver, thus representing paracrine or endocrine actions, respectively.

Finally, the activity of IGFs is regulated by six binding proteins (IGFBPs) in mammals and fish (223). Although some of the IGFBPs functions are IGF-independent, it has been clearly shown that they bind IGF-1 and IGF-2 with high affinity to prevent their degradation and to modulate their binding capacity to the IGF1R. *igfbp1*, *igfbp2* and their paralogs are mainly expressed in the liver and involved in the regulation of metabolism. *igfbp5* is readily expressed in muscle and associated to muscle growth, downregulated during fasting and upregulated during refeeding in trout in a similar manner than *igf-1* and *myogenin* (36), as well as its expression paralleled that of *igf-2* in a model of sustained exercise in gilthead sea bream (220). Due to the presence of numerous *igfbp* paralogs in the teleost genome, the functions of IGFBPs remain elusive. For a comprehensive overview of teleost IGFBPs, the reader is referred to the review by Garcia de la serrana and Macqueen (223).

### 4.5 Interleukin-6

IL-6 is a cytokine produced by immune cells, mesenchymal stem cells (MSCs), endothelial cells and fibroblasts (224). In addition, IL-6 was one of the first myokines identified in mammals due to its strong increase in muscle after exercise (225), leading to an increase in blood IL-6 levels (225, 226) that in turn, stimulates hepatic glucose production and lipolysis. In fish, IL-6 was identified in several species including rainbow trout (227) and zebrafish (228) but *il-6* expression in muscle has only been observed in zebrafish and very recently, in gilthead sea bream (200). Although the literature on fish IL-6 focuses on its functions in the immune system, transcriptomic analysis of muscle after exercise failed to observe increased *il-6* expression in zebrafish (221). Therefore, the function of IL-6 as a myokine in fish remains to be determined.

### 4.6 Others myokines

Although the number of identified myokines continues to increase in mammals, data in fish is still very scarce. For instance, the functions of irisin (229), myonectin (230) or the leukemia inhibitory factor (LIF) (231) on the crosstalk between fish adipose, muscle and/or bone tissues is still unknown arguing in favor of future work on this direction.

## 5 Osteokines

Bone is a specialized connective tissue composed of cells and a mineralized extracellular matrix (ECM). The skeleton serves as attachment of the muscle for locomotion, provides mechanical protection for internal organs, and constitutes a reservoir for hematopoietic stem cells and minerals contributing to calcium homeostasis. In mammals, it has been recently shown that the bone can act also as an endocrine organ through the secretion of specific hormones called ‘osteokines’ (232, 233). Evidence over the past decades have identified at least three bone-derived molecules showing endocrine functions. Osteocalcin and lipocalin-2 (LCN2) are secreted by osteoblasts and primarily involved in the control of energy metabolism and homeostasis (234), while fibroblast growth factor 23 (FGF23) is produced by osteoblasts and osteocytes and regulates phosphate homeostasis in the kidney (235). In this review, we will present the existing information about these bone-derived cytokines in fish.

### 5.1 Osteocalcin

Osteocalcin (a.k.a. bone Gla protein) is a small (45-50 amino acids) secreted molecule belonging to the vitamin K-dependent protein family and containing 3-4 γ-carboxyglutamic acid (Gla) residues, through which it interacts with calcium and hydroxyapatite. Produced by osteoblasts, osteocalcin is among the most abundant (10-20%) non-collagenous proteins found in the ECM of bone and dentin of most vertebrates, from bony fish to mammals (236). In fact, the appearance of osteocalcin seems to have paralleled hydroxyapatite-containing bone structures development, since it is not found in elasmobranchs, whose skeleton is composed of calcified cartilage (237). The mature γ-carboxylated form of osteocalcin has been associated with bone formation and mineralization, and it is required for the correct maturation of the hydroxyapatite crystals in mammalian bone (238). On the other hand, the uncarboxylated osteocalcin has been recently identified in mammals as a hormone regulating glucose metabolism, fat mass, angiogenesis, as well as male reproduction (239–241). Osteocalcin induces the production and secretion of insulin by pancreatic β cells, and promotes adaptation to exercise by stimulating the use of glucose and fatty acids by the skeletal muscle (242–244). In addition, osteocalcin controls cognition, and seems to be necessary for developing the acute stress response (189). However, none of these functions outside the skeleton have been postulated for a fish osteocalcin yet.

The *osteocalcin* gene originated before the division of the Teleostei and the Tetrapoda. In fish, the first *osteocalcin* characterized was that of the gilthead sea bream (245). Later on *osteocalcin* was identified in meagre (*Argyrosomus regius*), Adriatic sturgeon (*Acipenser naccarii*), common carp, pufferfish, medaka, Senegalese sole (*Solea senegalensis)*, white sea bream (*Diplodus sargus*) and rainbow trout among others (246–249). The gene structure and primary sequences of teleosts’ osteocalcins show many conserved features with those of other vertebrates, especially in the central region of the molecule (250). Conserved elements include two invariant cysteine residues that form a single disulfide bond in the mature molecule, as well as three γ-carboxyglutamic acid residues in the Gla domain, key to facilitate binding of osteocalcin to hydroxyapatite (251, 252). Moreover, during the characterization of osteocalcin from the meagre, a fourth carboxylation was reported (250). This feature was identified in other fish osteocalcins through multiple-sequence analysis; as well as in mammals, although no post-translational modification has been reported in those; therefore, its relevance remains to be elucidated. In any case, all known osteocalcins encode polypeptide chains as pre-pro-precursors with well-conserved features, suggesting the assemblage of a similar functional three-dimensional structure. Hence, mature osteocalcin binds strongly to hydroxyapatite to control crystals maturation, but also, a small fraction of osteocalcin (i.e., unbound) is released into the circulation, serum osteocalcin levels being considered the most sensitive marker of bone formation (240, 253).

Although most fish species only have one *osteocalcin* gene as in mammals, the existence of a second *osteocalcin* has been reported in some teleosts including zebrafish, rainbow trout, or Atlantic cod (*Gadus morhua)* (247, 254). Comparative analysis of the mature peptides revealed a high sequence conservation, suggesting that both isoforms may have the same function concerning bone mineralization. Indeed, this second osteocalcin is characterized by a large acidic and serine-rich pro-domain that perhaps, may be highly phosphorylated thus, potentiating its calcium binding properties (247). This second *osteocalcin* is thought to have arisen from WGD in the Teleostei lineage about 400 million years ago (247, 255). Moreover, in rainbow trout, the reported presence of paralogs for this second *osteocalcin* is in agreement with the salmonid-specific genome duplication (256).

In terms of tissue expression, fish *osteocalcin* as in other vertebrates is located only in mineralized bone tissues, including branchial arches, jaw, vertebra, scales and teeth (245, 257). Osteocalcin is associated to osteoblast-like cells and its content in fish bone is not dependent on the origin of the tissue, since similar levels have been found in fish with either cellular or acellular bone (i.e., devoid of entombed osteocytes). However, lower levels of *osteocalcin* mRNA have been reported in scaleless fishes such as channel catfish (*Ictalurus punctatus*), probably due to their need of using bone as the primary source of calcium for homeostasis instead of the scales as other fish species (258). Furthermore, as previously observed in mammals (259, 260), *osteocalcin* mRNA levels vary depending on the stage of development, as it is only present in calcified bone structures also in fish (245, 248, 261). In gilthead sea bream, *osteocalcin* appearance at around 30 days post-hatching coincides with the onset of skeleton mineralization (245), and the levels of expression at that time are greatly induced in response to increased incubation temperature during embryogenesis (262), which can be related with accelerated growth and the appearance of skeletal abnormalities. Indeed, inverse correlation between malformation severity and *osteocalcin* levels have been reported in fish (263–265). These evidences are supported by the knockout mice study in which *osteocalcin* deficiency led to higher bone mass by increased and accelerated bone formation (266).

Osteocalcin functions in mammals seem to be mediated through GPRC6A, a G protein-coupled receptor family C group 6 member (267, 268). In fish, this receptor was first identified *in silico* in relation to the olfactory epithelium and vomeronasal organ in several species (269). More recently, the expression and functionality of GPCR6A as amino acid receptor and sensor in the gastrointestinal tract of rainbow trout has been demonstrated (270), as in mammalian cells (271); nevertheless, its potential involvement mediating the role of *osteocalcin* in fish bone or other extra-skeletal tissues like muscle and adipose remains to be elucidated.

### 5.2 Lipocalin-2

LCN2 (a.k.a. neutrophil gelatinase-associated lipocalin or siderocalin), belongs to the lipocalin family of small secreted proteins (160-200 amino acids), which contains up to 50 members generated after WGD and specific gene duplications (272). Lipocalins share a common folding pattern resulting in a tertiary structure with eight-stranded antiparallel β barrels that enclose a hydrophobic substrate-binding pocket (273). Lipocalins have many different functions depending on their ligands (e.g., retinol, steroids, odorants, pheromones, siderophores, etc.).

Specifically, LCN2 is an iron-sequestering protein, first described in mammals as secreted by neutrophils, and mostly involved in innate immunity functioning as an antimicrobial protein (273–275). The expression of *lcn2* in rodents is also strongly induced in cartilage, embryo-derived hypertrophic chondrocytes and myocytes after stimulation with bacterial LPS, showing an inflammatory response during development (276). In humans, *lcn2* is highly expressed among others in the liver and adipose tissue, and circulating LCN2 has been identified as an inflammatory marker closely related to obesity and its metabolic complications (277). More recently, bone-derived LCN2 has been identified, and demonstrated that it influences energy metabolism by activating anorexigenic signaling pathways in the brain (278). These actions of LCN2 occur through binding to the melanocortin 4 receptor at the level of the hypothalamic paraventricular and ventromedial neurons.

In fish, a recent phylogenetic study of the lipocalin family, showed six members, being the lipocalin-type prostaglandin D synthase the evolutionary precursor of LCN2. In triploid crucian carp, a lipocalin named 3nLcn2 was recently described, showing a lipocalin domain and moderate to high levels of sequence identity with other fish lipocalins, although low when compared with mammalian sequences (279). Furthermore, 3nLcn2 presented antimicrobial activity against bacterial infection, especially exerting a protective effect at intestinal level, thus playing a role in immune defense in fish, as previously described in mammals. However, LCN2 bone-secretion or function as an osteokine have not been described yet for any fish species.

### 5.3 Fibroblast growth factor 23

FGF23 is in mammals, primarily expressed and secreted from the bone and related to phosphorous regulation (280), although it has been also proposed to act controlling body calcium homeostasis (281).

FGF23 is a 32 kDa protein, phylogenetically grouped with members of a FGFs subfamily that act as hormones or systemic factors interacting with specific receptors in the presence of members of the Klotho family of proteins. Indeed, FGF23 binds to Klotho, which encodes a type I membrane β-glycosidase-like protein that is an essential cofactor for FGF23 binding to FGF receptors (280).

In teleosts, *fgf23* and *αklotho* have been cloned in zebrafish among other species. Phylogenetic analysis showed as expected, that zebrafish *fgf23* is more closely related to *fgf23* from other fish species such as the rainbow smelt (*Osmerus mordax*) and the spotted green pufferfish, sharing 63 and 57% identity, respectively, than to the human gene (282). In adult zebrafish, *fgf23* is specifically expressed in the corpuscles of Stannius (282), which are endocrine glands that lie in close proximity to the nephron and are thought to be involved in calcium homeostasis, and *αklotho* is expressed in the kidney. *fgf23* expression is stimulated by ambient water with a high calcium level. Overexpression of *fgf23* in zebrafish lead to inhibition of calcium uptake by downregulating the expression of the epithelium calcium channel (ECaC) suggesting that FGF23 functions as a hypocalcemic hormone (283). Besides, a previous study also using zebrafish, reported that FGF23 regulates phosphate homeostasis (284). Interestingly, *fgf23* and *αklotho* mutations cause shortened lifespan in zebrafish, with early onset of behavioral and degenerative physical changes, and a specific calcification of vessels throughout the body (285). Altogether, these studies suggest a significant role of FGF23 and Klotho regulating fish mineral balance and aging-related processes, similarly as it occurs in mammals.

## 6 New approaches to specific analysis of tissue interactions

Other complementary studies that aim to decipher cellular interactions have been developed in mammalian models, including cells co-cultured, to try understanding *in vitro* how they communicate *in vivo* through contacts, secretions or both (286). For instance, adipocytes cultured in 2D as freshly isolated from tissues or immortalized cell-lines, evidenced some of these interactions: endocrine and paracrine mainly.

Indeed, due to adipose tissue evolution (175) and numerous adipose depots (visceral, subcutaneous, intra-muscular, brown…), adipocytes from endotherms were cultured with various cell types like those from blood vessels (287), cartilage (288), immune tissues (289), intestine (290) or even cancers (291). Herein, we focus on adipocyte-muscle co-cultures. Floating and fragile mature adipocytes used in co-cultures are rare as compared to cell-lines such as 3T3-L1, mouse fibroblasts that differentiate into adipocyte-like cells and the mouse myoblast cell line C2C12, themselves preferred to others (292). As an illustration for reciprocal dialogs among these cells: 3T3-L1 suppress muscle differentiation in C2C12 myocytes (293), while differentiated C2C12 inhibit proliferation and differentiation of 3T3-L1 adipocytes (294). Pre-adipocyte models, developed *in vitro* to provide differentiating adipocytes within 2 weeks of culture, did not resemble fully mature adipocytes with their large unilocular lipid droplet (295), but eased the co-culture in several species. Papers reporting on pre-adipocytes and muscle cells described different kinds of interactions and effects depending on the cell ratio, cell densities or length of co-culture time.

Studies with intra-muscular adipocytes in co-culture are rare, even though from their location they would be ideal partners to crosstalk with muscle MSCs. As an example, intra-muscular pre-adipocytes (IMPA) from chicken muscle impeded MSCs differentiation and promoted lipid deposition (26), proliferating MSCs inhibited IMPA differentiation and non-proliferating MSCs accelerated IMPA differentiation and lipid storage (296). Last, but not least, fibro-adipogenic progenitors, identified as multi-potent progenitors within the muscle, are highly influenced by the environment in their lineage choices and, conversely, influence muscle homeostasis by their secretions (297).

Based on single-cell or -nucleus sequencing, multiple adipocyte subtypes were recently reported (298, 299), opening the avenue to co-cultures with dedicated subpopulations depending on the cell-cell communications to be explored within adipose tissues or with distal organs (300). In addition, a renewed interest for the ECM: composition, physics and function (301) paves the way to the culture of fat cells or progenitors with autologous ECM, *in vivo* or *in vitro* derived (302).

Concerning other cell types, adipocyte stem cells stimulate 2D-co-cultured chondrocytes in a way that may contribute cartilage repair (303), whereas mature adipocytes modulate osteoclast differentiation (304), through the secretion of growth factors or adipokines: clear illustrations of an active bone-muscle crosstalk in (305). In the limits of our knowledge, no such co-cultures have been reported in fish species so far.

Thereafter, 3D-cultures appeared attractive to work out as a model since they include cell-cell contacts and so, putative juxtacrine signals (292, 306, 307). As an example, 3D *in vitro* structures were proposed for muscle/bone co-cultures using established cell lines for muscle (murine C2C12) and bone (human TE85 (308);). In optimal cases however, cultured cells look as closely as possible to those inside the tissues and keep this phenotype as long as possible to signal properly to the other cell types. For example, membrane mature adipocyte aggregate cultures helped maintaining adipogenic expression and long-term cultures with no functional loss, so that subcutaneous or visceral adipocytes retained depot-specific expression after 14 days (289) while mature adipocytes are reputed fragile and alive *in vitro* for only a few hours or days.

Although co-culture systems in fish remain to be developed, transgenic lines appeared in zebrafish that helped following *in vivo* adipocyte differentiation processes or adiposity dynamics (309), through the imaging of adipocyte- or lipid droplet-specific labels (fabp4-EGFP (310);; plin2-TOMATO (311);). Together with the use of a fasting-refeeding experiment (fabp11-EGFP (312);) or a high fat diet (311) these lines helped reporting on the metabolic regulations that impact on adipose tissues.

Furthermore, extracellular vesicles (EVs) isolated from various biological fluids (blood, lymph for instance) or cell culture media, mediate important communication between donor and recipient cells. In fact, EVs are released by a wide variety of cell types and are found in all organisms investigated so far (313). Recipient cells catch them by endocytosis, ligand binding or membrane fusion (313). From donor cells, large EVs or microvesicles (MVs) form by budding of the plasma membrane and vary in diameter from 250 to 500 nm. Small EVs or exosomes (40-100 nm) form inside the cell by fusion of intraluminal vesicles with the plasma membrane, and share with EVs common cargos such as proteins, lipids, RNAs (314). However, cargos of MVs or exosomes can also be selective, regulated by physiological or pathological states (315), their release thus resembling paracrine/endocrine signals. EVs secreted by the adipose tissue affect bone remodeling (via lipid transfer, circulating miRNAs or EV-linked-adipokines (316);). And, a reciprocal EV-based crosstalk between muscle and bone has also been reported (317), highlighting the role of such vesicles, conserved among eukaryotes (318). Interestingly, zebrafish embryo-expressing fluorescent proteins associated with exosomes (e.g., CD63-phluorin) have been demonstrated to be a relevant model to study endogenous EVs *in vivo* to unravel fundamental aspects in EVs physiology and their role in inter-organ communication (319, 320).

In mammals, interplays among cytokines and/or exosomes, also contribute to the metabolic situations altered in obesity or improved by exercise. As an example, metabolic organs defined as adipose tissue, gut, skeletal muscle and liver, can communicate with macrophages infiltrated into the adipose tissue in obesity cases (321). Similarly somehow, exercise-induced soluble factors among which adipokines, myokines, osteokines contribute to redistributed energy, appetite control, fat loss and reduced systemic inflammation (322, 323). Therein, the adipose tissue appears as an endocrine organ that interacts with others through cross talking with the endothelial cells of the blood vessels (287). Similar interplays might well be active in fish species as well, but few papers have addressed the question except one dealing with feeding, appetite regulating mechanisms, and energy homeostasis (324), proposing teleost fish as models to understand mammalian dysregulations.

## 7 Conclusions

To conclude, our current knowledge about adipokines, myokines, and osteokines physiologies relies, in a great part, on different findings regarding peptide structure, receptors, and expression patterns in a variety of fish species and tissues. The scenario is very complex in teleost fish, since these molecules are described in a wide variety of tissues and sometimes, different isoforms are identified. In addition, profiles of tissue distribution are different in many cases from those in mammals, probably associated with the specific fish physiology. That happens with some adipokines, which in fish, are rather expressed in liver, or in skeletal muscle, which are important organs of lipid deposition. Subsequently, the denomination of these factors as adipo-, myo- or osteo-kines could be more diffuse in fish.

Here we provide evidence of the existence of crosstalk between tissues from studies on the autocrine, paracrine, and endocrine effects of these cytokines and more specifically, findings from null mutations or knockouts of specific genes (i.e., *mstn*, *lepr*). Nevertheless, the importance of this communication between adipose, muscle and bone tissues during fish development, or along the life cycle, is not known yet.

Another unresolved question is the identity of the secretomes from these tissues in basal conditions or during fish exercise, fasting or aging, in order to adjust the whole organism’s homeostasis. The identification of molecular actors involved in inter-tissue communication opens new avenues of knowledge and strategies to improve growth and ameliorate possible metabolic disorders or skeletal anomalies in cultured fish.

Adipose tissue, muscle and bone are intimately linked, both in forms and in functions from embryogenesis to growth and development. Therefore, bone-muscle, muscle-adipose tissue, and bone-adipose tissue communication must be bi-directional with a complicated network of cytokines involved in such crosstalk. Research in this aspect could be greatly helped by the development of new experimental tools to determine whether these cytokines are responsible of a particular pathway of communication between tissues. New approaches such as the co-culture of different cell types, the study of exosomes or even the development of organoids will thus improve the understanding of the mechanisms underlying tissues crosstalk in fish.

## Author contributions

IH, EC, J-CG, and IN contributed to conception and design of the review. ER-M, SB-P, and VG prepared the table and figures. IH, EC, ER-M, J-CG, and IN wrote sections of the manuscript. All authors contributed to manuscript revision, read, and approved the submitted version.
